# The Influence of Hyaluronic Acid Biofunctionalization of a Bovine Bone Substitute on Osteoblast Activity In Vitro

**DOI:** 10.3390/ma14112885

**Published:** 2021-05-27

**Authors:** Solomiya Kyyak, Andreas Pabst, Diana Heimes, Peer W. Kämmerer

**Affiliations:** 1 Department of Oral- and Maxillofacial Surgery, University Medical Center Mainz, 55131 Mainz, Germany; solomiya.kyyak@unimedizin-mainz.de (S.K.); diana.heimes@unimedizin-mainz.de (D.H.); 2 Department of Oral- and Maxillofacial Surgery, Federal Armed Forces Hospital, 56072 Koblenz, Germany; andipabst@me.com

**Keywords:** bone substitute, bovine, xenograft, oral regeneration, biofunctionalization, hyaluronic acid, osteoblasts

## Abstract

Bovine bone substitute materials (BSMs) are used for oral bone regeneration. The objective was to analyze the influence of BSM biofunctionalization via hyaluronic acid (HA) on human osteoblasts (HOBs). BSMs with ± HA were incubated with HOBs including HOBs alone as a negative control. On days 3, 7 and 10, cell viability, migration and proliferation were analyzed by fluorescence staining, scratch wound assay and MTT assay. On days 3, 7 and 10, an increased cell viability was demonstrated for BSM+ compared with BSM− and the control (each *p* ≤ 0.05). The cell migration was enhanced for BSM+ compared with BSM− and the control after day 3 and day 7 (each *p* ≤ 0.05). At day 10, an accelerated wound closure was found for the control compared with BSM+/− (each *p* < 0.05). The highest proliferation rate was observed for BSM+ on day 3 (*p* ≤ 0.05) followed by BSM− and the control (each *p* ≤ 0.05). At day 7, a non-significantly increased proliferation was shown for BSM+ while the control was higher than BSM− (each *p* < 0.05). The least proliferation activity was observed for BSM− (*p* < 0.05) at day 10. HA biofunctionalization of the BSMs caused an increased HOB activity and might represent a promising alternative to BSM− in oral bone regeneration.

## 1. Introduction

Presently, the demand for soft tissue and hard tissue regeneration is frequently increasing where bone is one of the most transplanted tissues because of a multitude of congenital or acquired diseases [1]. Nevertheless, the field of bone transplantation and regeneration faces limitations regarding infections, immunological reactions, failed osteointegration and graft resorption [2]. To avoid graft harvesting and to support a better and faster regeneration, numerous materials are combined to find suitable alternatives to autogenous bone grafts [3]. Bone substitute materials (BSMs) of a xenogeneic, an allogeneic and an alloplastic origin are well established and widely used as suitable alternatives in numerous fields of medicine [4,5,6,7]. In the range of craniomaxillofacial regeneration, BSMs can cover a wide variety of clinical indications such as alveolar ridge preservation and augmentation, sinus floor elevation and the bony reconstruction of congenital or acquired maxillofacial malformations and defects [8,9,10,11].

Xenogeneic BSMs of bovine origin are long-term established and widely spread [12]. The hydroxyapatite-based substance [13] is known for its biocompatibility, sufficient osteoconduction and low up to no resorption [14,15] and its similarity to human bone due to its microstructure [16,17] and crystalline phase [18]. In contrast to autogenous grafts, BSMs do not contain organic components such as osteogenic cells or growth factors such as BMP-2 (bone morphogenic protein-2) and a VEGF (vascular endothelial growth factor) and they also may not contain collagen structures and fibers, enabling an osteoconductive and inductive regenerative potential in autogenous grafts. Thus, different BSM preparation methods and processes could affect the regeneration and surface characteristics of xenogeneic BSMs [19,20,21,22]. Accordingly, BSM sintering under a temperature >1000 °C seems to remove all organic compounds, thereby excluding an immune reaction and disease transmission and increasing crystallinity and volume stability [13,21,23,24,25]. Furthermore, it has been observed that even after a high temperature treatment, xenogeneic BSMs preserve their surface characteristics and a good biological performance [20,21,22,26]. Additionally, the carbonate content of high temperature treated hydroxyapatite stimulates human osteoblast (HOB) attachment and proliferation [27]. Nevertheless, it appears that BSMs may not be able to perform with an equal regenerative potency compared with autogenous grafts caused by the acellular and inorganic matrix. To overcome this limitation, BSM biofunctionalization has become more and more popular and has been tested in different ways. Recent studies analyzed combinations of BSMs with growth factors (e.g., BMP-2, VEGF) and PRF (platelet-rich fibrin). The findings of these studies illustrated that such biofunctionalized BSMs have the potency to accelerate and increase bone formation and vascularization as characteristic hallmarks of fast and sufficient bone regeneration [28,29,30,31,32,33]. As BSM modification with growth factors is technically challenging and restricted by legal requirements in most countries, further substances might be of interest for BSM biofunctionalization.

Hyaluronic acid (HA) is one of the largest components of the extracellular matrix. It is a long polysaccharide composed of macromolecules of many repetitive units of glucuronic acid and N-acetyl-glucosamine, remaining the same within all species [34,35,36]. It is stated that HA may regulate cell proliferation, differentiation, adhesion and gene expression [37]. These characteristics have aroused interest in HA in cutaneous research, cartilage grafting [38,39] and even bone reconstruction [34,40,41]. Thus, Kawano et al. reported that HA enhanced BMP-2 osteogenic bioactivity [35]. It has been discussed that HA retards bone resorption and osteoclast genesis through its receptor, CD44 [42]. HA may demonstrate lubricity under peculiar circumstances [43] and has been studied to have a bacteriostatic effect [44]. Sasaki et al. suggested that high molecular HA serves as a retainer for osteoinductive growth factors, thus stimulating osteogenic cell differentiation [45]. In addition, HA may positively influence angiogenesis and (neo-) vascularization because of its possible effects on endothelial cells, thus in turn indirectly stimulating new bone formation [45,46].

Different variations of HA molecules and their possible influence on tissue formation have been discussed. Guo et al. suggested that the molecular weight of HA strongly influences pro- and/or anti-inflammatory reactions of various tissues as far as peculiar angiogenic processes [47,48]. For example, Pilloni et al. observed that HA of a high molecular weight is dose-independent and not able to present any significant effects on bone formation [49]. However, further studies showed opposite findings [50]. This led to a significant interest in HA as an additive to different polymers and BSMs in bone engineering and regeneration.

Thus, the objective of this study was to analyze the influence of a commercially available BSM with (+) and without (−) HA biofunctionalization on viability, migration ability and the proliferation rate of human osteogenic cells. The zero hypothesis claims that this HA biofunctionalization has no influence on osteoblast activity.

## 2. Materials and Methods

### 2.1. Bovine Bone Substitutes

A commercially available xenogeneic bone substitute material (BSM−) of bovine origin (cerabone^®^, granularity: 1–2 mm; botiss biomaterials GmbH, Zossen, Germany) and a commercially available BSM with an HA modification (BSM+; cerabone^®^ Plus, granularity: 0.5–1 mm; botiss biomaterials GmbH) were used.

### 2.2. Cell Culture

Commercially available human osteoblasts (HOBs) were applied in the present study (HOB; PromoCell, Heidelberg, Germany). A HOB medium was supplemented with Dulbecco’s modified Eagle’s medium (DMEM; Gibco Invitrogen, Karlsruhe, Germany), fetal calf serum (FCS; Gibco Invitrogen), streptomycin (100 mg/mL; Gibco Invitrogen), dexamethasone (100 nmol/L; Serva Bioproducts, Heidelberg, Germany) and L-glutamine (Gibco Invitrogen). The HOBs were cultured according to standard protocols in an incubator at 37 °C, 95% humidity and 5% of CO_2_. Reaching a 70% confluence, the HOBs were passaged using 0.25% trypsin (Seromed Biochrom KG, Berlin, Germany) until passage five. The plates were filled with 100 mg BSM+/− together with 5 × 10^4^ HOB per well, respectively (27 wells per group, two groups). The plates with HOBs alone served as a negative control group (overall 27 wells). A further incubation was performed under the same conditions as by cell passaging. The measures were conducted in three time points in triplicate for each group and for each time point (days 3, 7 and 10; overall 81 wells).

### 2.3. Cell Viability

To analyze the HOB cell viability, CellTracker staining (Life Technologies, Thermo Fisher Scientific, Darmstadt, Germany; catalog number: C34552) was performed on days 3, 7 and 10. Red dye was prepared and used according to the manufacturer’s protocol. After the removal of the culture media, red dye was added into the wells. After 30 min, the red dye was removed and a serum-free medium was applied. The wells were further incubated for 30 min at 37 °C. After the removal of the serum-free medium, a fluorescence microscope (BZ-9000; Keyence, Osaka, Japan) for cell imaging was used where one image per well in ten-fold magnification was conducted. The cell quantification was managed by means of ImageJ software (ACTREC, Navi Mumbai, India) [51] by the following steps: the conversion of the images into grayscale, the correction of the background by image subtraction, automatic thresholding for cell structure extraction from the background and the final calculation of the percentage area fraction (%). The measures were carried out in triplicate for each group and for each time point by three time points (on days 3, 7 and 10; overall 9 wells per group).

### 2.4. Cell Migration

The cell attachment was measured by means of a scratch wound assay. A scratch wound was performed at the bottom of the wells with a sterile pipet tip (p200; Gilson, Middleton, USA) on days 3, 7 and 10 [52]. Immediately after the scratch, a fluorescence microscope (BZ-9000; Keyence, Osaka, Japan) for cell imaging was used. Twenty-four hours later, red dye staining was obtained for preparing images with the aforementioned microscope (one image for each well, 9 wells per group, ten-fold magnification). An area of migrated cells into the gap was quantified by the percentage area (%) using ImageJ software as described before [51]. The measures were carried out in triplicate for each group and for each time point (three time points).

### 2.5. Cell Proliferation

The proliferation activity was measured by a 3-(4,5-Dimethylthiazol-2-yl) -2,5-diphenyltetrazolium bromide (MTT) assay on days 3, 7 and 10. An MTT solution (200 µL, 2 mg/mL) was applied to the cell culture medium in the wells followed by 4 hours of incubation at 37 °C. After the removal of the culture medium and washing up by phosphate buffered saline, a lysis buffer (Isopranol (49 mL) with 2N NCl (1 mL; 1 mL per well) was added. The measurement was performed without the BSM in separate wells using a fluorescence microplate reader with a wavelength of 570 nm (Versamax; Molecular Devices, San Jose, CA, USA). The measures were carried out in triplicate for each group on days 3, 7 and 10 (overall 9 wells for each group).

### 2.6. Statistics

The mean values were interpreted into a standard error of the mean (SEM) in the cases of parametric data and into median values for non-parametric data. The numbers were rounded (to two decimal places). The normal distribution was defined by a Shapiro-Wilk test. In the case of a normal distribution, to compare two subgroups a two-sided Student’s *t*-test for paired samples was applied. In the case of non-normal distributions, a Mann-Whitney test was used. For a comparison of all subgroups, a Kruskal-Wallis rank sum test was performed. *p*-values ≤ 0.05 were considered to be significant. Data were illustrated with bar charts including error bars.

## 3. Results

### 3.1. Cell Viability

**Figure 1:**
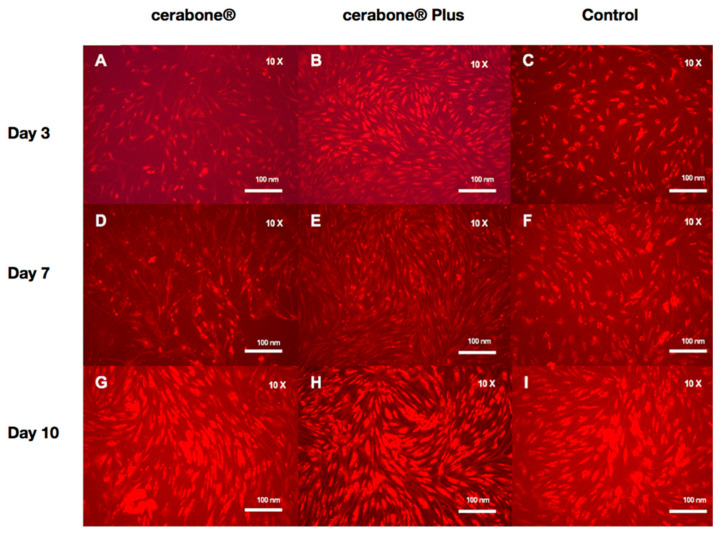
Fluorescence imaging (red cell tracker) in groups with BSM− (cerabone^®^), BSM+ (cerabone^®^ Plus including HA) and the control (HOB alone) on days 3 (**A**–**C**), 7 (**D**–**F**) and 10 (**G**–**I**).

**Figure 2:**
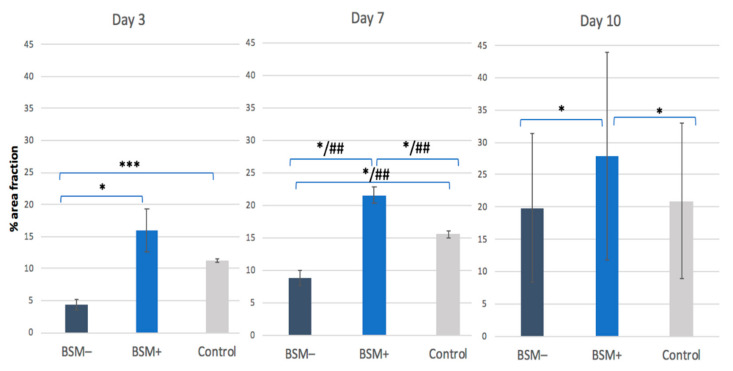
Cell Viability. Percentage area fraction (%) of fluorescence-stained HOBs at a ten-fold magnification for BSM− (cerabone^®^), BSM+ (cerabone^®^ Plus including HA) and the control (HOB alone) on days 3, 7 and 10. * = *p* ≤ 0.05, *t*-test; *** = *p* ≤ 0.0001, *t*-test; ## = *p* ≤ 0.05, KWT.

**Figure 3:**
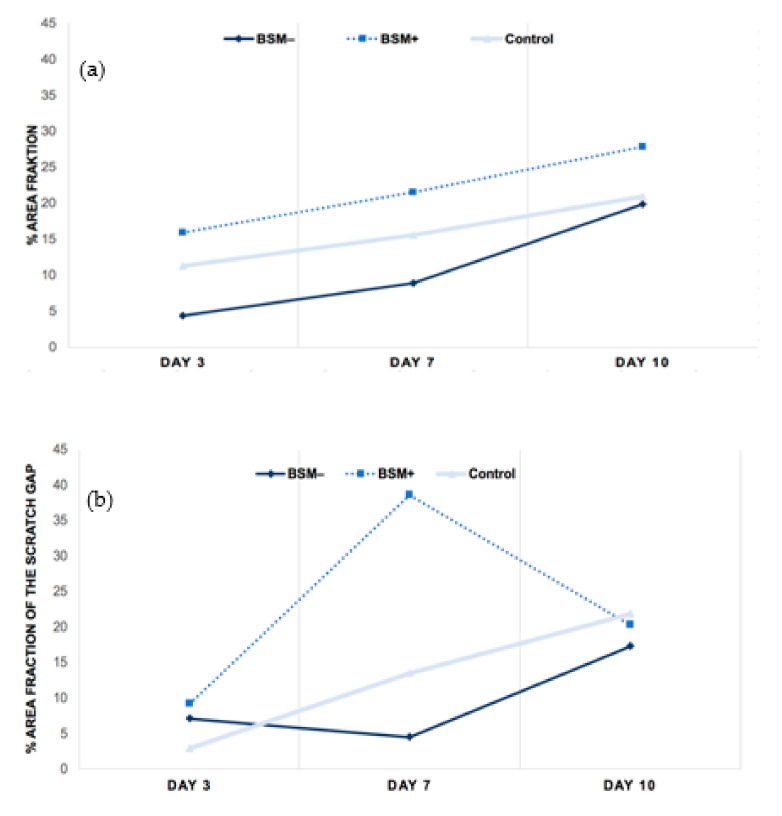

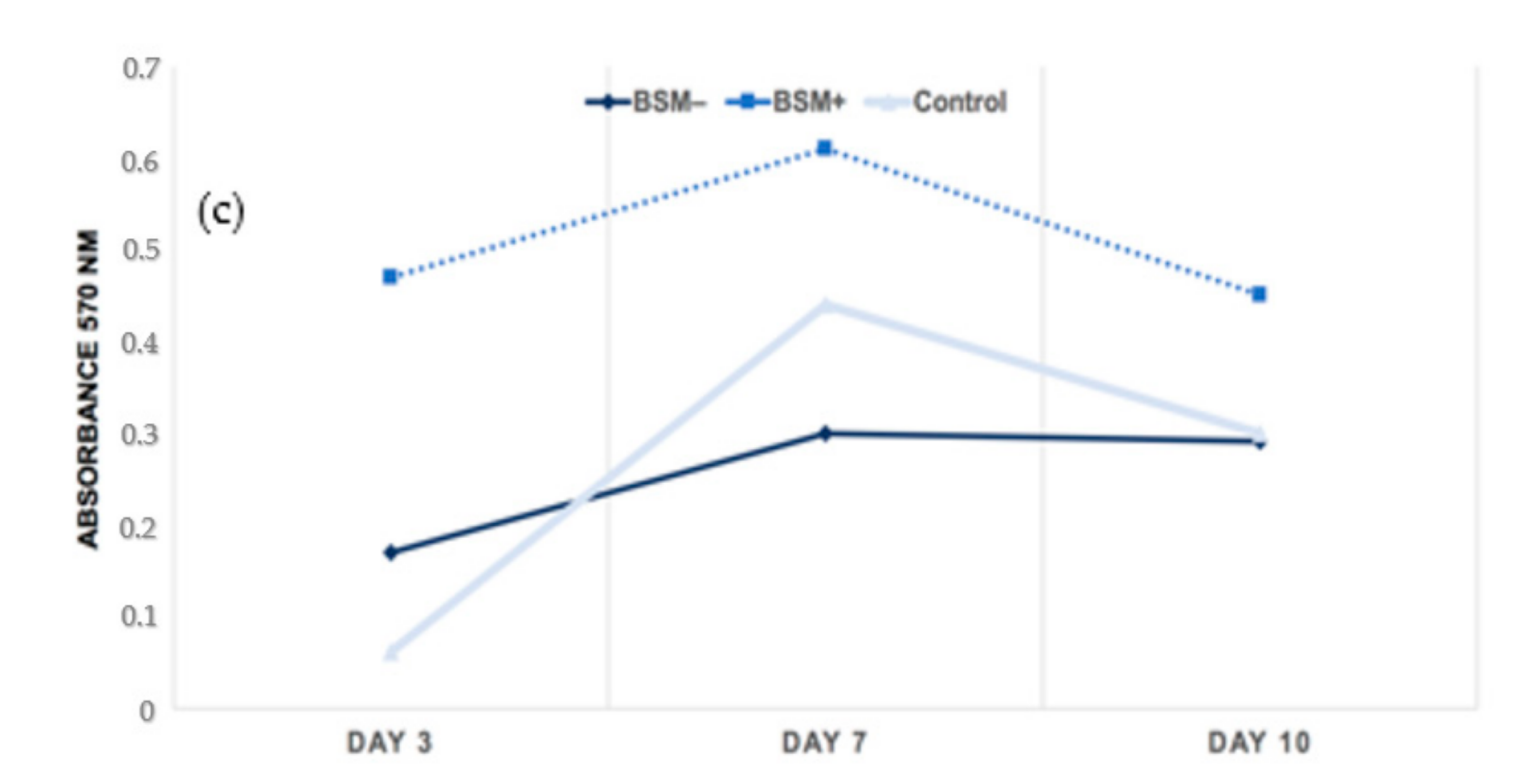
Tendency through the period of 3, 7 and 10 days within groups BSM− (cerabone^®^), BSM+ (cerabone^®^ Plus) and the control (HOB alone). (**a**) Cell viability, (**b**) migration ability, (**c**) proliferation rate.

On day 3, the highest cell viability was observed for BSM+ when compared with BSM− (*p* = 0.028, *t*-test) and the control (*p* = 0.24, *t*-test). The cell viability of the control group was significantly higher than BSM− (*p* < 0.001, *t*-test) On day 7, the highest cell viability was seen for BSM+ compared with BSM− (*p* < 0.001, *t*-test; *p* < 0.05, KWT) and the control (*p* = 0.014, *t*-test; *p* < 0.05, KWT) followed by the control when compared with BSM− (*p* = 0.006, *t*-test; *p* < 0.05, KWT). At day 10, the cell viability of BSM+ was significantly higher when compared with the controls (*p* = 0.004, *t*-test) and BSM− (*p* = 0.002, *t*-test) (Table 1, Figure 1 and Figure 2). Although the cell viability values for BSM+ were the highest of all groups through the whole period, the greatest tendency to increase was observed in BSM− in which the cell viability raised almost five times compared with BSM+ and the control with approximately two times (Figure 3a).

### 3.2. Cell Migration

**Figure 4:**
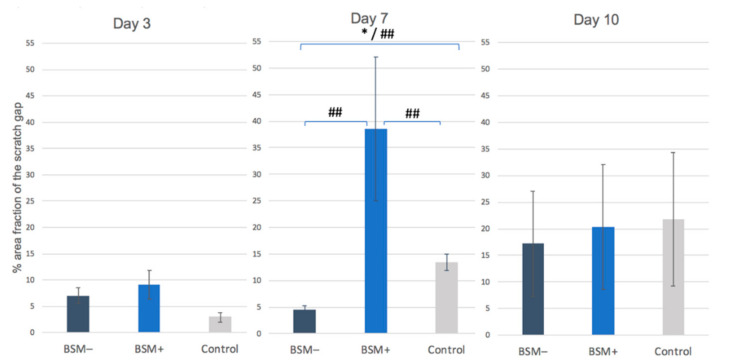
Migration ability: Percentage area fraction of the scratch gap (%) of fluorescence-stained HOBs at a ten-fold magnification for BSM− (cerabone^®^), BSM+ (cerabone^®^ Plus including HA) and the control (HOB alone) on days 3, 7 and 10. * = *p* ≤ 0.05, *t*-test; ## = *p* ≤ 0.05, KWT.

On day 3, the highest cell migration rate was found for BSM+ followed by BSM− and the control (each *p* > 0.05, *t*-test). On day 7, the highest value was observed for BSM+ (*p* < 0.05, KWT). The controls showed a significantly increased proliferation rate when compared with BSM− (*p* = 0.007, *t*-test). On day 10, the best wound closure was observed for the control followed by BSM+ and BSM− (*p* > 0.05 each, *t*-test) (Table 2, Figure 4). The migration ability in BSM+ increased from day 3 to day 7 by five and a half times and then decreased almost two times until day 10, being on day 10 almost on the same level with BSM− and the control group (Figure 3b).

### 3.3. Cell Proliferation

**Figure 5:**
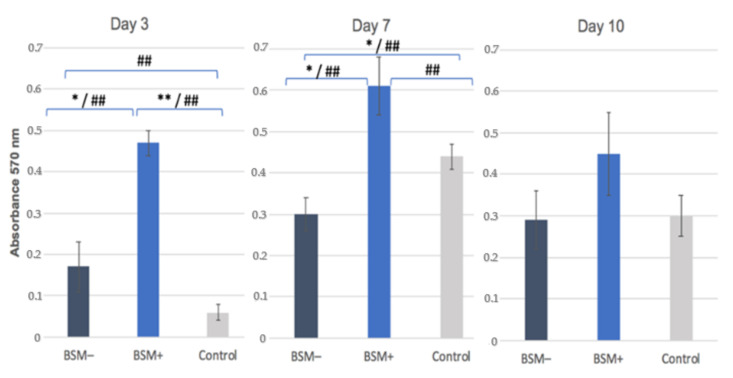
Cell Proliferation: MTT assay, absorbance at 570 nm for BSM− (cerabone^®^), BSM+ (cerabone^®^ Plus including HA) and Control (HOB alone) on days 3, 7 and 10. * = *p* ≤ 0.05, *t*-test; ** = *p* ≤ 0.001, *t*-test; ## = *p* ≤ 0.05, KWT.

On day 3, the highest cell proliferation was observed for BSM+ in comparison with BSM− (*p* = 0.011, *t*-test; *p* < 0.05, KWT) and the control (*p* < 0.001, *t*-test; *p* < 0.05, KWT) followed by BSM− and the control (*p* < 0.05 each, KWT). On day 7, an increased proliferation rate was shown for BSM+ in comparison with BSM− (*p* = 0.019, *t*-test; *p* < 0.05, KWT) and the control (*p* < 0.05, KWT) while the control demonstrated increased values compared with BSM− (*p* = 0.046, *t*-test; *p* < 0.05, KWT). On day 10, the least proliferative activity was measured for BSM− (*p* > 0.05, MWT). Here, the highest proliferation rate was demonstrated for BSM+ (*p* > 0.05, MWT) (Table 3, Figure 5). The groups generally showed a tendency to increase up to day 7 and decrease until day 10. The highest raise rate was observed in the control on day 7. However, BSM+ stayed far on the top throughout the whole period (Figure 3c).

## 4. Discussion

This in vitro study analyzed the effects of HA in combination with commercially available BSMs of bovine origin on human HOB cell viability, migration ability and proliferation rate. The overall findings demonstrated a significant benefit of HA biofunctionalization of BSMs on the above-mentioned HOB cell features responsible for bone regeneration. In brief, the modification of bovine BSM with HA significantly increased the biological activity of HOBs in comparison with the same BSM alone. The cell viability presented a smooth increase through the whole period where BSM+ stayed distinctly the highest of all groups. HA additivity activated the migration ability on days 3, 7 and 10. The cell proliferation in its turn was significantly affected on day 7 and presented only a slight difference among groups on day 3 and day 10.

In a previous study, we evaluated different commercially available BSMs of bovine origin in regard to their biological effect on human HOBs. Here, the high temperature (>1200 °C) sintered bovine BSM, which was included in the present study whether alone or in combination with an injectable PRF, seemed to have the best effects on HOB cell viability, metabolic activity and gene expression of alkaline phosphatase (ALP), osteonectin and BMP-2 when compared with other BSMs of bovine origin prepared at lower temperatures [29]. Hence, the aforementioned BSMs of bovine origin commercially modified with HA or pure were included in the present study. According to our results, the combination of HA manufactured by bacterial fermentation and bovine BSMs presents an increase in the biological activity of HOBs in comparison with the same BSM alone. Accordingly, the cell viability in all groups presented a smooth increase throughout the whole period where they stayed distinctly the highest in groups with HA modification. Moreover, HA biofunctionalization activated the proliferation rate of HOBs on days 3, 7 and 10. The cell proliferation in its turn was significantly affected on day 7 and presented only a slight difference among groups on days 3 and 10. Our findings, that HA positively affects HOB bioactivity, were in accordance with other in vitro and in vivo studies although, to the best of our knowledge, there are no in vitro studies dealing with information about the effects of HA in combination with BSMs of bovine origin on HOBs. Kawano et al. concluded that HA enhances the osteogenic activity of HOBs in vitro via the down-regulation of BMP-2 antagonists and the phosphorylation of extracellular signal-regulated kinase [35]. Thus, chemically cross-linked hyaluronan-based hydrogels with HA and BMP-2 demonstrated cancellous bone formation in ectopic sites after five weeks [53]. HA functionalization of a titanium surface seems to enhance HOB proliferation and alkaline phosphatase activity [54,55]. Furthermore, HA has been studied to modify the composition of the extracellular matrix, affecting its fibrillary and non-fibrillar components [56]. Sasaki et al. suggested that HA acts as a detent for growth factors even enhancing HOB activity [45]. Interestingly, HA and its side groups happen to reduce bacterial adhesion and prevent biofilm formation [57].

In spite of intensive research in this area, there are no evident studies proving a HA-specific mechanism of interactions and pathways considering osteogenesis [58]. It has been reported that HA affects wound healing by enhancing the CD44 surface marker consequently activating early inflammation and cell migration into granulation tissue [59]. Due to the similarity with the extracellular matrix, HA seems to be biocompatible inducing a low immune response. Furthermore, it accelerates cell adhesion, migration and proliferation and, as a result, to some extent new tissue formation [60]. However, HA presents a low mechanical strength and a high degradation rate, thus being limited and requiring appropriate modifications [61]. A combination of HA with gelatin and alginate into a three-dimensional composite scaffold showed to be high load bearing without fractural deformation [62]. Mathews et al. presented a scaffold with a chitosan-collagen-HA ratio of 1:1:0.1 in which lower HA concentrations and more uniform pores seemed to enhance HOB differentiation-promoting effects [63]. Furthermore, HA appears to be capable of encapsulating bioactive factors by cross-linking [64,65]. Nevertheless, the general process of HA cell bioactivation, due to its complexity, especially including osteogenesis, is still unclear [58]. Presently, the effect of HA as an enhancer of the biological properties of a synthetic scaffold, an activator of osteogenesis and as a vector for osteoinductive substances is approved [66].

It is known that HA combined with BSM+ is of a bacterial origin non-cross linked high molecular weight hyaluronic acid (h-HA) with a molecular mass of 1.9–2.1 MDa. However, it belongs to the limit of our study that the amount of it added to the BSM was not given. It has been reported that the molecular weight of HA is greatly decisive regarding the effect on the biological activity of cells and pro-inflammatory characteristics [45,67]. However, there is no consensus in the literature regarding the ideal constitution and concentration of HA for better bone regeneration [68]. Thus, Boeckel et al. observed a decrease in HOB viability under presence of HA and referred this not to chemical composition but rather to the molecular weight of HA [68]. The same findings were found in other studies [49,69,70]. Hence, it was suggested by that h-HA positively alters the cellular parameters of HOBs and influences peculiar inflammatory mediators, acting as an adjustor of HOB biological capacities [71]. Furthermore, Agarwal et al. demonstrated that h-HA in comparison with a low molecular one (l-HA) presented a significantly increased osteogenic differentiation of HOBs based on an upregulation of ALP, collagen and EM mineralization as well as the effects of l-HA, in its turn, on HOB proliferation and adhesion [50].

Another limitation of our study was sample staining using the Cell Tracker 5-chloromethylfluorescein diacetate for cell viability, which also permeates dead cell membranes. However, stained live cells are >100-fold brighter than dead cells and could be easily distinguished from the dead population [72]. It also belongs to the limits of the study that bone substitutes of two different particle sizes were compared: 0.5–1 mm versus 1–2 mm. However, the difference was not significant and may not have affected the results [73,74,75]. The critical difference was studied to be between the particles of less than 0.4 mm and more than 1 mm [73]. However, another study contradicted this statement, concluding that particles of 0.1–0.3 mm and 0.5–0.7 mm were not significantly different in terms of their osteogenic potential [74]. Another study suggested that the granularity was not of a significant relevance but was rather dependent on the clinical defect size. It seems that the microstructure characteristics of the material rather than its granularity plays an important role [75].

The implementation of HA in combination with bone substitute materials may be very promising to overcome any limitations in the soft and hard tissue regeneration. HA modified BSMs have the advantage of being classified as commercially available medical devices ready to use. Further in vitro and in vivo studies of HA in combination with BSMs of different origins will carve out the significance of dosage and the molecular weight of HA in bone engineering as far as there are no specific mechanisms of interactions and pathways considering HA involved in osteogenesis [58]. Clinical trials will focus on visible benefits such as the bone regeneration capacity and long-term stability in vivo.

## 5. Conclusions

HA biofunctionalization of BSMs enhancing the viability, migration ability and proliferation rate of human osteogenic cells on days 3, 7 and 10 might be able to accelerate and improve bone regeneration and might represent a promising alternative to native BSMs.

## Figures and Tables

**Table 1:** Cell Viability. Percentage area fraction (%) of fluorescence-stained HOBs at a ten-fold magnification for BSM− (cerabone^®^), BSM+ (cerabone^®^ Plus including HA) and the control (HOB alone) on days 3, 7 and 10. The mean values are for parametric data and the median values are for non-parametric data.

	Day 3		Day 7		Day 10	
	Mean Value	SEM	Mean Value	SEM	Mean Value	SEM
BSM−	4.41	±0.82	8.85	±1.14	19.86	±11.47
BSM+	15.92	±3.38	21.55	±1.32	27.84	±16.08
Control	11.22	±0.22	15.57	±0.54	20.9	±12.07

**Table 2:** Migration ability: Percentage area fraction of the scratch gap (%) of fluorescence-stained HOBs at a ten-fold magnification for BSM− (cerabone^®^), BSM+ (cerabone^®^ Plus including HA) and the control (HOB alone) on days 3, 7 and 10. The mean values are for parametric data and the median values are for non-parametric data.

	Day 3		Day 7		Day 10	
	Mean Value	SEM	Mean Value	SEM	Mean Value	SEM
BSM−	7.01	±1.49	4.51	±0.75	17.19	±9.93
BSM+	9.15	±2.74	38.57	±13.47	20.3	±11.72
Control	2.91	±0.92	13.46	±1.59	21.81	±12.59

**Table 3:** Cell Proliferation: MTT assay, absorbance at 570 nm for BSM− (cerabone^®^), BSM+ (cerabone^®^ Plus including HA) and the control (HOB alone) on days 3, 7 and 10. The mean values are for parametric data and the median values (*) are for non-parametric data.

	Day 3		Day 7		Day 10	
	Mean Value	SEM	Mean Value	SEM	Mean Value	SEM
BSM−	0.17	±0.06	0.3	±0.04	0.29	±0.07
BSM+	0.47	±0.03	0.61	±0.07	0.54 *	-
Control	0.06	±0.02	0.44	±0.03	0.3	0.05

## Data Availability

Data are available on request.
